# What’s the Matter with the A.M.A.?

**Published:** 1911-09

**Authors:** 


What's the Matter With the A. M. A.?—*The Texas State Journal of Medicine* says: "There were in the neighborhood of 3000 physicians in attendance upon the Los Angeles meeting in June." My! "how she has shrunk." Time was when 6000—7000—attended—out of a membership of 30,000. After all the drumming, horn-blowing and pull of passenger agents—3000—or about 10 per cent were induced to go.

---

Married at Rockport, Texas, July 20, Dr. Jno. Wesley Crutcher to Owene Elizabeth Peeler, daughter of Mr. and Mrs. A. J. Peeler of Houston. "At home" at Mineral Wells, Texas, after August 5.

---

If a patient with the signs and symptoms of chronic appendicitis is unduly anemic and has lost weight, be prepared for the possibility of a more serious lesion, e. g., neoplasm, ileocecal tuberculosis.—*American Journal of Surgery.*

---

Toothache or Earache Remedy.—Take dram vial and fill with pure water; then drop a tablet of atropin, 1-100 gr., into it. When dissolved it is ready for use. Drop two or three drops into the tooth or ear, as the case may be, and plug with absorbent cotton. Repeat every thirty minutes till relieved.—*Medical Brief.*

